# The complexity of human subjective experience during binocular rivalry

**DOI:** 10.1093/nc/niaf004

**Published:** 2025-02-28

**Authors:** Cemre Yilmaz, Laura Pabel, Elias Kerschenbauer, Anja Ischebeck, Alexandra Sipatchin, Andreas Bartels, Natalia Zaretskaya

**Affiliations:** Department of Psychology, University of Graz, Graz, ST 8010, Austria; Department of Psychology, University of Graz, Graz, ST 8010, Austria; Department of Psychology, University of Graz, Graz, ST 8010, Austria; Department of Psychology, University of Graz, Graz, ST 8010, Austria; BioTechMed-Graz, Graz, ST 8010, Austria; Center for Integrative Neuroscience, University of Tubingen, Tubingen, BW 72076, Germany; Center for Integrative Neuroscience, University of Tubingen, Tubingen, BW 72076, Germany; Department of Psychology, University of Graz, Graz, ST 8010, Austria; BioTechMed-Graz, Graz, ST 8010, Austria

**Keywords:** binocular rivalry, consciousness, perception, transition, vision

## Abstract

Our subjective experience of the sensory information is rich and complex. Yet, typical cognitive and perception psychology paradigms reduce it to a few predefined discrete categories, like yes/no answers or the Likert scales. In the current study, we examined the complexity of subjective visual experience during binocular rivalry, a major experimental paradigm used to study conscious visual perception and its neural mechanisms. Binocular rivalry occurs when the two eyes are presented with two different images that cannot be fused into a uniform percept. As a result, the conscious perception alternates between the two images with brief transition phases in between. Fifty-two subjects viewed binocular rivalry produced by pairs of stimuli with different visual information (images, orthogonal gratings, or moving dots). After each rivalry period, they indicated how many different transition types they perceived and described their perception of each transition type. Using content analysis, we identified 20 unique categories over all subjects, sessions, and stimuli. On average, participants reported 2–3 unique transition categories for each visual stimulus combination. The categories were consistent for each observer over time but varied across participants and stimulus content. Our results show that perceptual transitions during binocular rivalry appear in different forms and depend on the specific visual stimulus content that induces rivalry. Our findings have implications for neuroimaging studies of binocular rivalry, which may yield different results depending on the exact experience of transitions. They also demonstrate how the complexity of subjective visual experience may be underestimated in traditional perception paradigms.

## Introduction

Our conscious experience of the visual input is rich and complex. Viewing even a simple object, such as an apple, evokes a myriad of impressions: outline, color, texture, and 3D shape. However, common research practices tend to reduce the complexity of subjective experience to predefined categories. For example, in a near-threshold detection task, observers are asked whether they perceived a stimulus presented briefly, with a yes/no response. Similarly, when presenting an ambiguous stimulus such as the Necker cube or the Rubin’s vase illusion, the task is to report perception by choosing one of the two possible interpretations. Such simplifications are hardly compatible with complex and dynamic nature of our perception.

Binocular rivalry is one of the key paradigms for studying conscious experience and its neural basis. It occurs when we view two different images separately through the two eyes. The images cannot be fused, and the conscious percept of the observer alternates between perceiving either image with brief transition periods in between, even though nothing changes physically. Periods of transitions in binocular rivalry are used to identify the neural correlates of internally-generated changes in perception (Lumer et al. 1998), sparking a debate about the contribution of the frontoparietal network to conscious visual experience (Carmel et al. 2010, Zaretskaya et al. 2010, Frässle et al. 2014, Brascamp et al. 2015, Gelbard-Sagiv et al. 2018, Weilnhammer et al. 2021, Kapoor et al. 2022). Periods of transitions in binocular rivalry are thus an important phenomenon for studying the neural correlates of consciousness.

In recent years, there is an increasing interest in perceptual transitions during binocular rivalry beyond consciousness research. For example, Klink et al. (2010) showed that the prolonged exposure to rivalry stimuli might lead to longer transition periods and shorter periods of exclusive percepts. This pattern of results is thought to reflect experience-dependent plasticity in the synaptic connections between monocular neurons, which are typically involved in mutual inhibition. With repeated exposure to binocular rivalry input, the inhibition may be ceasing as the visual system learns to fuse dissimilar images, giving rise to longer periods of seeing a mixture of the two. Furthermore, transition durations positively correlate with symptoms of autism spectrum disorder (Robertson et al. 2013, Karaminis et al. 2017, Dunn and Jones 2020) as well as attention-deficit hyperactivity disorder (Casanova et al. 2013, Amador-Campos et al. 2015, Jusyte et al. 2018). For both disorders, transition lengthening has been interpreted as a reduction in inhibitory interactions. All these studies show that transitions in binocular rivalry reflect basic processes behind brain function and dysfunction.

The majority of previous studies regarded transitions in binocular rivalry as a single perceptual event in addition to the two image percepts (Cao et al. 2016, Roy et al. 2017, Riesen et al. 2019). However, the existing literature clearly implies that there is more than one transition type. Specifically, Yang et al. (1992) described “piecemeal” transitions, consisting of local patches of either of the stimuli, and “superimposed” transitions, appearing as if one semi-transparent stimulus is overlayed on top of the other one. Interestingly, experience-dependent plasticity through repeated exposure to binocular rivalry has been shown to affect specifically the superimposed, but not piecemeal transitions (Klink et al. 2010). Another study showed that the superimposed transitions were longer than piecemeal transitions in response to the contrast modulation (Skerswetat et al. 2020). Increasing evidence for the argument that the different conditions influence the durations of superimposed and piecemeal transitions (Sheynin et al. 2020, Skerswetat et al. 2022) suggests different underlying mechanisms. Furthermore, transitions can appear as a “traveling wave”, in which the conflicting image starts appearing from one edge of the dominant percept and slowly spreads throughout the whole visual field (Wilson et al. 2001, Knapen et al. 2007). Despite the evidence that transitions may appear in different forms, and may reflect different underlying neural mechanisms, the exact appearance of transitions in binocular rivalry has remained outside of the research focus. By systematically examining the appearance of transitions and factors that affect them, we can reveal the mechanisms of conscious visual perception, basic visual mechanisms such as binocular fusion and plasticity, as well as mechanisms underlying various neuropsychiatric disorders.

In the current study, we investigated the phenomenology of transitions in binocular rivalry. We extended the classical binocular rivalry task, where participants report which image they perceive, by adding a questionnaire about their subjective experience of transitions. We also varied the visual information that induces binocular rivalry by including complex and simple, static and dynamic stimuli. On average, participants perceived transitions in at least three different ways for each stimulus pair. Across all stimulus pairs, sessions and participants, we could identify 20 unique transition types. Our results demonstrate that subjective experience during binocular rivalry is more complex than previously thought.

## Methods and materials

The main hypotheses, experimental procedures, and data analysis strategy were preregistered prior to the start of the data collection on AsPredicted.org (https://aspredicted.org/2BP_X1Y).

### Observers

A total of 52 observers were recruited for the study. After applying the exclusion criteria (see below), a total of 41 observers who were between 18 and 61 years old (mean = 29, SD = 8.4, 24 F, 17 M) were used in the final analysis. We confirmed that the sample size was sufficient with an *a priori* power analysis for our primary hypothesis (reported number of transitions larger than 1) using G*Power (Faul et al. 2007, 2009). The following settings were used: one-sided one-sample *t*-test, assumed medium effect size, 80% power, *P* value of .05. The power analysis yielded a required sample size of 27 to be sufficient to reject the null hypothesis. All the observers gave their written informed consent and agreed with data protection policy prior to participating. The experiment was approved by the ethics committee of the University of Graz.

### Screening and exclusion criteria

Prior to the main experiment, observers underwent a screening procedure to make sure they fulfilled the inclusion criteria. They first performed Freiburg Visual Acuity Test $FrACT_{10}^{2022 - 04 - 26}$ (https://michaelbach.de/ot/FrACT10/capp/index.html; Bach 1996) and then a Random Dot V Stereo Test (http://www.neuro-o.se/CritVis/cVis2.html#3DV). Both tests were presented on an LCD monitor (1920 px × 1080 px, diagonal display size: 22 inches, vertical refresh rate: 60 Hz, viewing distance: 230 cm, Samsung, Seoul, Korea) using a Bluetooth numpad (Bluetooth v.5 numeric keypad with multifunction 22 keys, CSL-Computer, LLC, Langenhagen, Germany) as a response device in a dark room.

Observers were excluded if they had low visual acuity (logMAR < 0.0) and/or poor stereovision (stereoacuity < 30 arcsec) or strong eye dominance (> 0.8 dominance of one eye in each block). Eye dominance was determined based on binocular rivalry responses in the first session, and was defined as the median percept duration of one eye divided by the sum of the median percept durations of both eyes (Ding et al. 2018). Furthermore, the accuracy of observers in their report of conscious perception during the replay session was used as a measure of response reliability (see “Binocular rivalry and replay analysis” section for details). We analyzed the scores for potential outliers by applying 1.5 interquartile range rule (Wolpaw et al. 2000). We excluded five observers as outliers in accuracy. Nine observers were excluded in total based on the above criteria (visual/stereo acuity: 3, eye dominance: 1, response accuracy: 5).

### Binocular rivalry and replay stimuli

All stimuli for the main experiment were generated using MATLAB 2017b (MathWorks, Inc., Natick, MA, USA) and Psychtoolbox (Brainard 1997, Pelli 1997, Kleiner et al. 2007) on a Linux Ubuntu 20.04 LTS computer, and presented on a gamma-corrected LCD display (1920 px × 1080 px, diagonal display size: 24 inch, vertical refresh rate: 60 Hz, viewing distance: 65 cm, Asus, Inc., Taipei, Taiwan). The monitor had a maximum luminance of 90.40 cd/m^2^ and its brightness setting remained at 100% for all observers.

Binocular rivalry was achieved using a first-surface 4-mirror stereoscope (ScreenScope SA200LCD, Stereo Aids, Albany, AL, USA). Throughout all experiments and conditions, stimuli were presented foveally and were surrounded by a checkerboard ring, which was identical for the two eyes to aid stable vergence. The checkerboard ring extended between 2.35 and 9.38 degrees (dg) of eccentricity. A red fixation dot at the center (0.22 dg in diameter) was also displayed superimposed on both stimuli.

**Figure 1:**
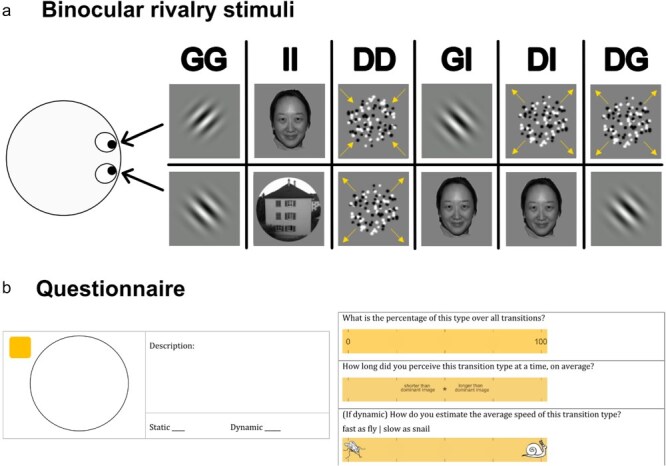
Experimental design. (a) Observers were presented with blocks of binocular rivalry stimuli and asked to press either the left or the right arrow button to report their dominant percept in each block. They were instructed not to press any button during transition periods. Each block contained a pair of stimuli with different visual content: gratings (GG), images (II), dots (DD), grating-image (GI), dots-image (DI), and dots-grating (DG). (b) After every block, observers were first asked to state how many transition types they experienced. After this, they described their perception of each transition type (left panel) by writing a short text and making a drawing. These data were used for subsequent transition categorization using content analysis (see “Transition categorization” section for details). They also estimated the frequency, duration, and speed of each transition type using three scales (right panel).

We used three stimulus types commonly used in binocular rivalry studies (Fig. 1): (i) orthogonal static sinusoidal gratings tilted ±45 from the vertical orientation with a spatial frequency 2.04 cycles/dg within a Gaussian envelope, (ii) images of a face and a house, and (iii) dots moving inward or outward radially with a speed of 0.59 dg/sec. Each frame had a dot density of 21.27 dots/dg and each dot had a 0.015 dg diameter. To prevent observers from tracking individual dots, we restricted the lifetime of each dot to 40 frames. For gratings, the contrast was set to 100% Michelson contrast, for images to 0.4 RMS contrast, and the contrast for the dots was set to 1 (half of the dots) or −1 (another half of the dots) Weber contrast. Each stimulus was 2.35 dg in diameter.

### Experimental design

The experiment consisted of three sessions completed on three separate days interleaved by 4–6 weeks. The interval between the first and second session was 32 ± 7 (mean ± SD) days and the interval between the second and third session was 35 ± 10 days. The task was the same in each session: observers were asked to fixate the central red dot and to report their perception of binocular rivalry by pressing two keys (either left or right arrow). They were asked not to press any keys when unsure of their perception (transition). Additionally, they were asked to pay attention to the appearance of transitions. The first two sessions included the rivalry task with six different stimulus pairs in a pseudorandom order: (i) gratings, (ii) images, (iii) moving dots, (iv) grating and image, (v) moving dots and image, and (vi) moving dots and grating, as illustrated in Fig. 1a. The second rivalry session was added to assess the consistency of observer’s reports over time. The third session was a “replay” condition in which the stimulus presentation was an imitation of rivalry reported by each observer in the first session. The replay task was used to measure observers’ response accuracy (see “Screening and exclusion criteria” section for details).

Each session consisted of six blocks with one stimulus pair per block. Each block consisted of 2 trials, each lasting 2 min, with 15 s breaks in between. The stimuli were swapped between the eyes after the first trial to decouple percept duration from eye dominance.

**Figure 2:**
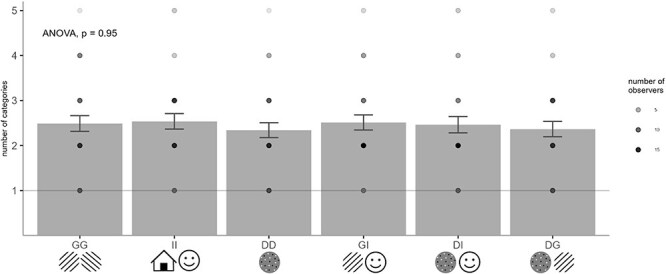
The number of categories reported by observers for each stimulus pair. Bar plots represent the sample average and the error bars represent standard error of the mean (SEM). The gray level of dots indicates the number of observers reporting that many transition categories. Observers reported significantly more than 1 category per stimulus pair, and the number of categories did not differ across stimulus pairs.

At the end of each block, the observers answered a questionnaire about the appearance of transitions (Fig. 1b). Using the questionnaire, they indicated how many transition types they perceived and were asked to describe each transition type that appeared in the corresponding block by writing a short text and/or drawing a simple sketch (see Fig. 2 for examples). There were no restrictions on the time spent on the questionnaire and no word limit. Each transition type had to be described on a separate sheet. Observers were allowed to use as many sheets as the number of transition types they indicated to have perceived in each block. After they described the appearance of all transition types in a block, we asked them to estimate their frequencies (what percentage of transitions appeared as described), durations (relative to the average duration of the dominant percept), and speeds (subjectively fast or slow) using a computer-based questionnaire generated with Octave 4.2.2 (Eaton et al. 2017) and Psychtoolbox on a Linux Ubuntu 20.04 LTS laptop (Model: P82G, Dell, Inc., Austin, TX, USA). In the questionnaire, they could provide an estimate of these values by clicking the position of a corresponding scale. This was done to additionally test for stimulus-dependent changes in these parameters (e.g. higher or lower proportion of transitions of a certain type for one stimulus pair vs. the other).

In the replay session, the responses of each observer in session 1 were used to create an imitation of rivalry with two transition types: abrupt and smooth transitions (see Jusyte et al. 2018 for details). The abrupt transitions were generated by presenting stimulus 1 which immediately switched into a superimposed transition (overlayed stimuli with 50% transparency) followed by stimulus 2. In a smooth transition, on the other hand, the transparency of stimuli changed gradually with one becoming opaquer and the other more transparent. The duration of transitions followed the duration of reported transitions in the first rivalry session. In each block, a trial with abrupt transitions was followed by a trial with smooth transitions. After each replay block, participants were presented with a questionnaire and were asked to describe transitions in the same way as during rivalry.

### Data analysis

#### Traditional binocular rivalry and replay analysis

Using observers’ key presses, we determined their eye dominance and excluded them if there was more than 0.8 or less than 0.2 left eye dominance (“Screening and exclusion criteria” section for details). Moreover, we computed median dominance duration (median key press duration irrespective of percept type) and median transition duration (median duration of periods when no button was pressed) for each stimulus pair in session 1. Previous studies reported that asymmetries in stimulus complexity may affect binocular rivalry dynamics, including the duration of transitions (Alais and Melcher 2007). To check if there is an asymmetry in dominance duration of the two stimuli in a pair, we compared the dominance durations of the two stimuli in a pair using a paired *t*-test.

For the replay analysis, we first plotted observers’ key presses together with the actual stimulus presentation times to visually check how well the observers responded to the physical stimulus changes. We found that seven observers confused the buttons in the block with gratings and pressed left arrow for right-tilted grating and right arrow for left-tilted one. We corrected their data to avoid inconsistency between observers. After this, we calculated the accuracy score for each observer, which was calculated at the level of perceptual epochs (events) as the following. First, a visual event was defined as either a percept or a transition. Then, we looked for a button press throughout the duration of that event. For a percept event, a button press of the corresponding stimulus, e.g. left button for house and right button for face, was considered correct. For a transition event, the absence of a key press was considered correct. For the smooth replay trials, transitions were defined as transparency values between 0.25 and 0.75 of stimulus 2. The overall accuracy was defined as the number of correct events divided by the total number of events.

The duration of perceptual transitions in binocular rivalry are known to follow a gamma or a gamma-like distribution (Levelt 1967, Brascamp et al. 2005). Recently, it has been shown that transition durations in binocular rivalry also follow a gamma-like distribution, similar to the dominance durations (Riesen et al. 2019). To confirm this in our data, we fitted a gamma distribution to the empirical distribution of dominance and transition durations after normalizing each participant’s durations by their mean using the *fitdistrplus* package in R (Delignette-Muller and Dutang 2015). We report the fitted parameters and goodness-of-fit (chi-squared test) for transitions and dominance periods.

#### Transition categorization

The drawings and the verbal descriptions of observers were processed using content analysis method, which is commonly used in the field of social psychology (Bengtsson 2016). Content analysis typically deals with a body of text such as an essay or an interview, termed “text unit”. The actual piece of text which undergoes categorization is called “coding unit”. Text unit and coding unit can be identical (e.g. an essay), or a coding unit may constitute a part of a text unit (e.g. a chapter of an essay). During the analysis of a coding unit, “themes” are deduced from the text. Themes summarize the coding unit, similar to keywords. Finally, the actual analysis guideline including all the steps until the assignment of categories as well as the set of categories to which coding units can be assigned is called “coding system”. Applying this framework to the analysis of transition descriptions, we considered the questionnaires for individual stimulus pairs as text units and the descriptions of the individual transition types as coding units. The descriptions of one observer were considered together, forming a “context unit”. This was important in order to account for the individual differences in how observers describe their perception. For example, the term “quick transition” was used by some observers to describe a brief transition that was perceived as a third image, whereas others used it to describe an immediate switch from one dominant percept to another. We defined the themes considering such individual differences. Therefore, themes were consistent across observers. Themes allowed us to standardize the vocabulary used by the actual subjects and to facilitate the categorization process. The raters considered only the themes while assigning the category for each individual coding unit at the next step of categorization.

The transition categorization was performed using the iterative categorization procedure (Neale 2016) as follows. First, the initial five complete datasets (data from five observers) were used by two raters, who already had experience with binocular rivalry to examine the coding units and define the themes. The raters checked whether the themes can be assigned to one of the four transition categories identified in our previous pilot experiment (“halves”, “piecemeal”, “superimposed”, and “traveling waves”) (Sipatchin et al. 2018). If a theme did not fit any of the four categories, the raters defined a new category. This categorization step was considered to be the “training” step and was performed by the two raters collaboratively. Second, the 2 raters proceeded with categorizing the coding units independently using data from additional 25 observers, defining new categories if necessary (total *N* = 30). At the end of this procedure, common categories of the two raters were identified and the reliability of the coding system was assessed by calculating Cohen’s Kappa (k = 0.43). In the third step, the two raters agreed on the final category assignment and on the coding system (category names and their abstract descriptions, correspondence between themes and categories). In the fourth step, we tested our coding system with the same 30 observers, but with a new rater. The datasets of the first five observers again served as the training data, in order to make sure that the third rater understood the coding system and the procedure. Feedback was given on categorization performance during training. The third rater then went on to categorize the data of the remaining 25 observers without feedback. To assess the reliability of the coding system, Cohen’s Kappa was calculated between the categories defined by the first/second rater and the third rater. The coding system was moderately reliable (Cohen’s Kappa = 0.51), which was considered sufficient for the third rater to proceed with categorization on his own (McHugh 2012). Another round of discussions to reach an agreement on the final set of categories followed and the coding system was updated to its final version. The third rater, who could not experience binocular rivalry due to a vision deficit but had theoretical knowledge about the phenomenon, proceeded with categorizing data of the remaining 11 observers alone using the final coding system and the categorization of the initial 30 observers was updated according to the final coding system. The whole procedure is schematically depicted in the Supplementary Figure S1. Our final coding system is presented in Supplementary materials.

Some transition descriptions appeared to be “composite” transition types; i.e. consisting of a combination of different “clear” types (e.g. superimposed piecemeal). Furthermore, some transition descriptions appeared to represent either static or dynamic version of the same percept (e.g. superimposed and dynamic superimposed). We nevertheless defined separate categories for composite, dynamic and static transitions, because observers themselves identified these as separate categories (i.e. described them as separate transition types on the questionnaire).

#### Statistical analysis

The rivalry parameters derived from the key presses, the number of perceived transition types indicated by the observers in the questionnaire and the results of the categorization procedure described above were used to perform statistical analysis. Statistical analysis was performed in RStudio 4.3.1 (PBC, Boston, MA, USA).

First, we tested if participants reported more than one transition type for each stimulus pair. For this analysis, we used the number of categories indicated by each observer in the questionnaire, comparing this value with one using a one-sample *t*-test. Since the number of categories reported by participants in two sessions was similar (Spearman rank correlation range over stimulus pairs: *R* = 0.42–0.67, p_fdr_ < 0.01), we only report results for session 1. To investigate the effect of stimulus pair on the number of reported categories, we performed a one-way repeated measures analysis of variance (ANOVA) with six levels. Note that this analysis is entirely independent from the categorization procedure and is based entirely on the number of transitions indicated by each participant in the questionnaire.

Second, we tested the effect of stimulus pair on the “appearance” of transitions. For this we examined transition categories identified for each subject and each stimulus pair. For this analysis, we transformed the data of each session into a 20 (categories) × 6 (stimulus pairs) presence–absence matrix. To test the consistency of observers’ categories over sessions, we used the presence–absence matrices of each session to calculate the Sorensen–Dice coefficient, which is widely used to quantify the similarity between two binary matrices. It is calculated by dividing twice the number of elements common to two matrices by the sum of the number of elements in both. The range is between 0 and 1, which can be translated into “totally different” to “identical” datasets. Since this analysis showed high similarity between sessions (Sorensen–Dice coefficient range over observers: 0.73–0.94), we only report results for session 1. To assess the impact of stimulus pair on transition appearance, we used the Cochran’s Q test, which is a categorical equivalent of a one-way repeated measured ANOVA, using two categories (present, absent). Using Cochran’s Q test, we compared the presence–absence of each category separately across stimulus pairs. We ended up with 19 Cochran’s Q tests showing the differences in transition categories across the stimulus pairs, correcting for multiple comparisons with false discovery rate (FDR) method. As a *post-hoc* test, we performed a pairwise McNemar’s test (a categorical equivalent of a paired *t*-test) on the categories showing a significant effect of stimulus pair.

To additionally visualize the differences in observers’ descriptions of transitions depending on the stimulus type, we used preprocessed themes pulled over subjects and categories based on stimulus type. Preprocessing included unifying spelling, removing stop words, stemming, and handling negations.

We also analyzed the subjective estimates of frequency, duration, and speed of each transition category to further investigate the effect of stimulus content on the features of categories. In rare cases, where observers reported two transition types which we then assigned to the same category (e.g. the side of the start of the traveling wave, the side on which the house was located relative to the face half), their frequency estimates were summed and speed and duration estimates were averaged. Since not all categories were reported by each observer, we selected the categories that were reported by at least 19 observers for each stimulus pair. The cutoff of 19 was determined by the power analysis with the following parameters: medium effect size, alpha level of 0.05, power of 80%, number of measurements: 6. Since our data violated the assumption of normal distribution, we performed a Kruskal–Wallis test to test the difference in frequency, duration, and speed estimates across the stimulus pairs, correcting for multiple comparisons with FDR method. For the significant Kruskal–Wallis test results, we performed *post-hoc* pairwise Wilcoxon signed-rank tests to compare the estimates across stimulus pairs.

Finally, we also performed a one-way ANOVA to test the effect of stimulus pair on the conventional binocular rivalry parameters (median percept duration, median transition duration). As a *post-hoc* test, we performed paired-sample *t*-tests. The *P* values for the *post-hoc* tests were corrected for multiple comparisons with FDR method.

## Results

### Observers report more than one transition category

We first tested whether observers perceived transitions in more than one way by statistically comparing the number of categories indicated in the questionnaire against one. Since the number of categories was consistent across sessions (Spearman rank correlation range over stimulus pairs: *R* = 0.42–0.67, p_fdr_ < 0.01), we report the results of session 1 only. Indeed, observers reported significantly more than one transition category for every stimulus pair (Fig. 2 and Table 1), with averages ranging between 2.58 and 2.95. This number did not change depending on the stimulus [F(5, 111.98) = 0.218, *P* = .95]. Note that the number of transitions used in this analysis are raw values as indicated by the observers in the questionnaire; i.e. prior to the categorization.

**Table 1.** The results of a one-sample *t*-tests against 1 to test if the observers reported more than one transition category per stimulus pair

Stimulus pair	No. of categories (mean ± SD)	*t* value	*P* value	Effect size (Cohen’s d)
Gratings (GG)	2.95 ± 1.18	10.57	<.001	1.65
Images (II)	2.80 ± 1.29	8.97	<.001	1.40
Dots (DD)	2.75 ± 1.37	8.18	<.001	1.28
GratingImage (GI)	2.88 ± 1.49	8.09	<.001	1.26
DotsImage (DI)	2.93 ± 1.39	8.91	<.001	1.39
DotsGrating (DG)	2.58 ± 1.16	8.74	<.001	1.37

### Transitions vary in appearance

**Figure 3:**
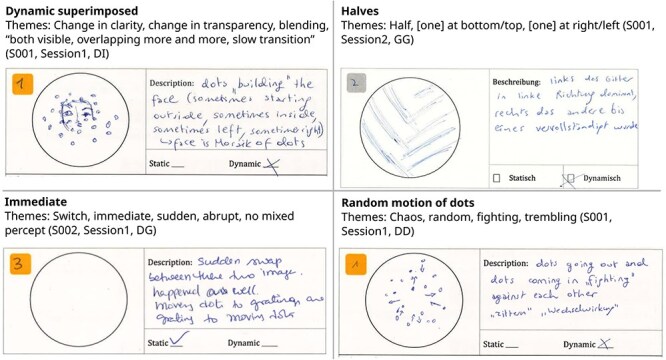
Example questionnaire responses, corresponding themes and categories from two representative observers.

To categorize participant’s descriptions of the transition types, we performed iterative content analysis (Neale 2016) with three independent raters. This analysis revealed a total of 20 unique transition categories over all stimuli, observers and sessions: cancellation, center/surround, circular motion of dots, circular wave, dispersing/gathering, dynamic piecemeal, dynamic superimposed, dynamic superimposed piecemeal, halves, immediate, partial cancellation, piecemeal, random motion of dots, superimposed, superimposed circular wave, superimposed dispersing/gathering, superimposed halves, superimposed piecemeal, superimposed traveling wave, and traveling wave. In all, 19 out of 20 categories were present in session 1. Example drawings and descriptions of observers, corresponding themes of the content analysis, and assigned categories are shown in Fig. 3 (see Supplementary materials for a complete list of categories and themes).

### Transition categories are consistent within and between observers

**Figure 4:**
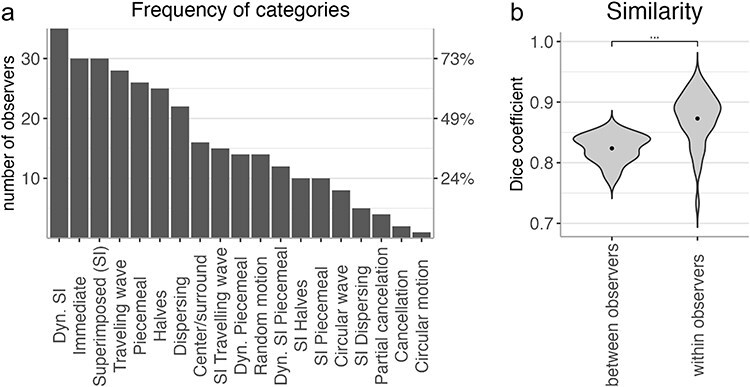
Frequency and consistency of transition categories. (a) The number of observers reporting each transition category in session 1. The *y*-axis shows the count (left) and percentage (right) of observers who reported this transition type for at least one stimulus pair. (b) Between- and within-observer similarity of reported categories, measured by the Sorensen–Dice coefficient. Between-observer similarity was calculated by comparing the categories reported by each observer with those of every other observer using Sorensen–Dice coefficient and subsequent averaging. Within-observer similarity was calculated by comparing the categories reported in the first and the second session. The error bars indicate SEM. ***: *P* < .001.

The categories identified for each observer were highly consistent across the two sessions (Sorensen–Dice coefficient mean ± SE: 0.87 ± 0.007). There were, however, some individual differences across observers, as illustrated in Fig. 4. While circular motion and cancellation transitions, for example, were reported by very few participants, dynamic superimposed, superimposed, immediate, traveling wave and dispersing transitions were reported by more than 50% of all participants (Fig. 4a). Accordingly, the pairwise similarity of categories between observers (Sorensen–Dice coefficient mean ± SE: 0.82 ± 0.003) was significantly lower than the session-to-session consistency within observers [t(40) = −10.33, *P* < .001, Cohen’s d = 1.44, Fig. 4b].

### Stimulus content affects transition appearance

**Figure 5:**
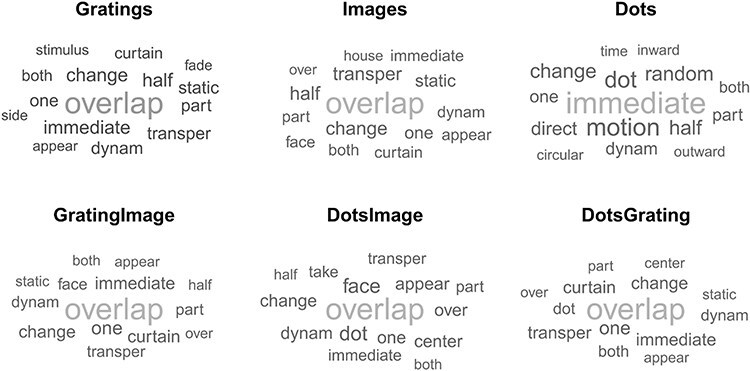
Themes in each stimulus pair. Word clouds representing transition perception for different stimulus types were created based on themes of the content analysis, after basic preprocessing.

To visualize the differences between stimulus types, we additionally used themes to create word clouds. Themes represent the standardized descriptions of perceptual transitions, an intermediate step prior to categorization. The size of words represents the relative frequency of terms across subjects (Fig. 5). The word clouds reveal that, although there are similarities across stimulus types (e.g. “overlap” appearing most frequently in 5 out of 6 stimulus types), there are also considerable differences, especially for the moving dots stimuli.

**Figure 6:**
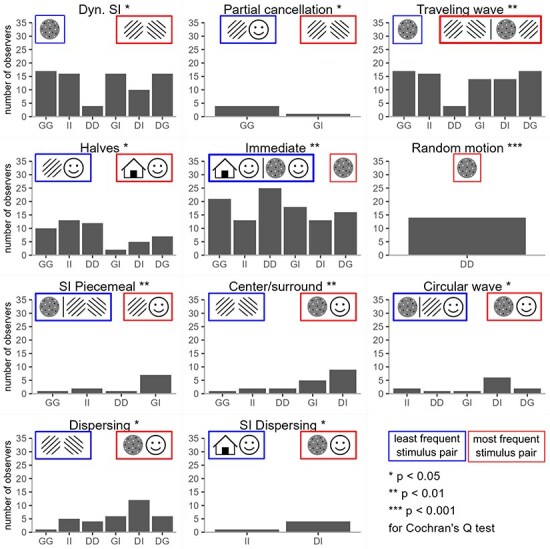
Frequency of each transition category for each stimulus pair in session 1. Asterisks indicate the significant effect of stimulus pair on the distribution of category frequencies according to Cochran’s Q test. *: p_fdr_ < 0.05, **: p_fdr_ < 0.01, ***: p_fdr_ < 0.001 (FDR-corrected) and icons illustrate stimulus pairs for which the examined transition category occurred most (red) or least (blue) frequently.

We further formally tested if the stimulus content influenced the appearance of transitions and found that some of the transition categories were more frequent for specific stimulus pairs (see Fig. 6). For example, random motion of dots appeared only for the Dots stimulus. In contrast, other categories (dispersing/gathering, dynamic piecemeal, dynamic superimposed, halves, immediate, piecemeal, superimposed, superimposed halves, and traveling wave) were observed for all stimulus pairs, but with varying frequency. Over all transition categories, stimulus pair had a significant impact on 11 out of 19 categories: center/surround, circular wave, dispersing/gathering, dynamic superimposed, halves, immediate, partial cancellation, random motion of dots, superimposed dispersing/gathering, superimposed piecemeal, and traveling wave (Fig. 6; see Supplementary Figure S2 for complete data on frequencies for each transition category). *Post-hoc* analysis revealed how exactly the stimulus content influenced transition appearance (Fig. 6, red and blue outlines). For example, immediate transitions (which is what binocular rivalry transitions typically assumed to look like) are most frequent for Dots stimuli and least frequent for Images or DotsImage combination. Another previously described transition category, the travelling waves, is, in contrast, least frequent for Dots stimuli, and most frequent for Gratings or GratingImage combination. Conceivably, random motion transitions were observed exclusively in the moving dot stimuli. Interestingly, transitions related to differences between center and periphery, such as center/surround, circular wave, dispersing, and superimposed dispersing, occurred most frequently for DotsImage stimuli, suggesting that they may be triggered by a competition between dorsal and ventral streams. Overall, this result shows that transition appearance might be directly influenced by the stimulus content (see Supplementary Figure S3 for data on all *post-hoc* pairwise comparisons).

**Figure 7:**
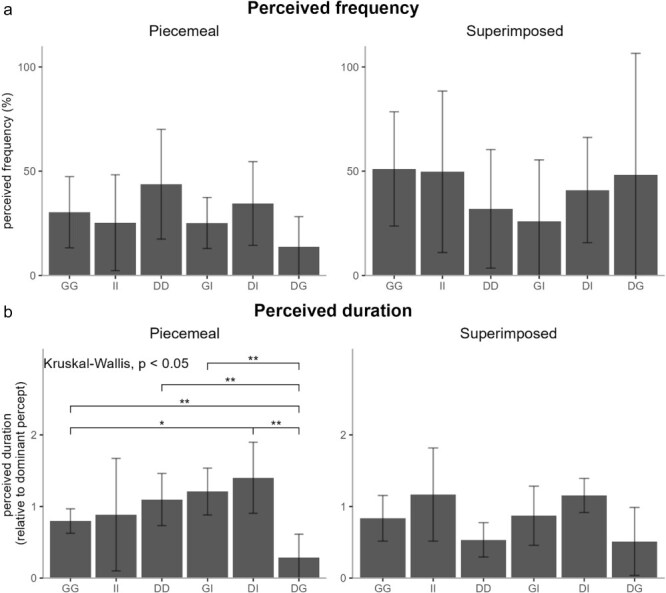
Questionnaire reports of perceived (a) frequency and (b) duration of each transition category across stimulus pairs. *: *P* < .05, **: *P* < .01, ***: *P* < .001 for *post-hoc* tests when Kruskal–Wallis test gave significant results.

For those transition categories that were frequent enough to be observed in at least 19 observers (number determined using power analysis) in every stimulus pair, we additionally tested if a more fine-scale analysis of observer-estimated frequency and duration of that transition category, as indicated by the observers in the questionnaire, is affected by the stimulus content (Fig. 7). This analysis revealed that the estimated duration of piecemeal transition was affected by the stimulus content. Piecemeal transitions for DotsGrating stimuli were perceived to be the shortest and for DotsImage stimuli to be the longest (Fig. 7b). We did not observe any effect on frequency of piecemeal transitions and no effect on superimposed transitions.

### Quantification of classical rivalry parameters

**Figure 8:**
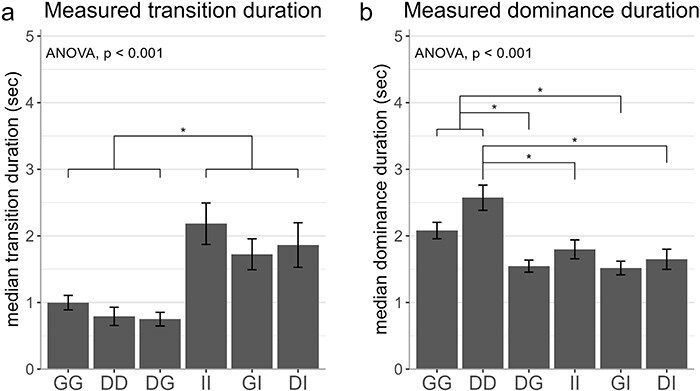
The effect of stimulus content on binocular rivalry parameters. We calculated (a) median transition duration and (b) median dominance duration for each stimulus pair in session 1. After ANOVA showed a significant effect of stimulus pair, we performed paired *t*-test to test for pairwise differences. *: *P* < .05 (FDR-corrected).

Finally, we also calculated the conventional rivalry parameters as reported by the key presses: median transition duration and median dominance duration. We also checked for the difference in dominance duration between the two stimuli in a pair, but none of the pairs showed a significant difference (all *P* ≥ .64). As illustrated in Fig. 8, transition duration was shorter than dominance duration (transition duration: 1.38 ± 0.10 and dominance duration: 1.86 ± 0.06, mean ± SD across observers). Both dominance duration and transition duration depended on the stimulus content [F(5, 109.35) = 7.63, *P* < .001 for transition duration and F(5, 110.99) = 7.20, *P* < .001 for dominance duration]. Importantly, the differences in the “measured” duration of all transition phases (Fig. 8a) cannot fully explain the differences in “perceived” duration of piecemeal [r(39): −1.02 to −0.02; *P*: .32 to .98 across six correlations] and superimposed [r(39): −0.05 to 0.36; *P*: .06 to .95 across six correlations] transitions (Fig. 7b), since the two measures are not correlated.

Moreover, we found that, similar to dominance durations, the normalized transition durations followed a right-skewed gamma distribution (all chi-squared tests *P* < .05, see Supplementary Table S1 and Supplementary Figures S4 and S5 for full results). This supports the notion that transitions may represent another perceptual state.

## Discussion

In this study, we demonstrated the variability of subjective experience during binocular rivalry. Our observers reported multiple types of intermediate percepts between the two images, and their appearance varied depending on the exact stimulus content used to induce binocular rivalry. We observed individual differences across observers, but also a high degree of consistency within observers over time, confirming the reliability of our results. The fact that intermediate percepts during binocular rivalry are diverse and variable has several important implications, which we discuss in detail below.

### Implications for behavioral studies

It has long been known that binocular rivalry transitions are not always immediate, and sometimes rather long. A recent study even suggested to treat transitions during binocular rivalry as a third perceptual state (Riesen et al. 2019). Additionally, with a more sophisticated response procedure using a joystick as well as objective measures of rivalry alternations, it has been shown that observers do not perceive one of the stimuli exclusively even in the periods of dominance (Naber et al. 2011). Subjective experience during binocular rivalry may thus be more complex than what is typically assumed and the boundaries between dominance and transition periods may be blurred, at least under some stimulus conditions. Our results further complicate this picture by demonstrating that transitions may represent not just one more, but several different perceptual states, which appear differently depending on whether moving dots, simple gratings, or complex objects are presented to observers. All these results yield a picture of binocular rivalry as a complex subjective experience, and it may be advantageous to use this complexity rather than disregarding it.

While there is a consensus that binocular rivalry transitions are not always instantaneous, evidence emerges that this may be the case for other ambiguous stimuli as well. For example, one study queried its observers about their perception of the ambiguous Rubin’s vase illusion and showed that a large proportion of individuals perceive the two percepts simultaneously (Filevich et al. 2017). Anecdotal evidence from our lab shows that transitions may appear in different forms for other ambiguous stimuli as well. During the presentation of the ambiguous apparent motion (Neuhaus, 1930), for example, in addition to instantaneous transitions some observers reported perceiving one of the dots moving horizontally and the other vertically. Therefore, we cannot exclude the possibility that different types of intermediate percepts exist for other bistable stimuli, and maybe even in other experimental paradigms used to probe conscious perception. Interestingly, a recent study on metacontrast masking also demonstrated the diversity of subjective visual experience of target and mask stimuli (Koster et al. 2020). Together with ours, these findings indicate that the complexity of subjective experience may be underestimated in several popular paradigms used to study conscious perception.

It is important to note that in contrast to more conventional ambiguous stimuli, binocular rivalry contains a strong low-level component (Blake and Logothetis 2002, Tong et al. 2006). This low-level component is thought to arise from interocular competition between groups of monocular neurons in V1, which are linked via inhibitory connections (Wilson 2003). For this reason, apart from being a key paradigm for probing conscious visual perception, binocular rivalry has been used to probe different aspects low-level visual processing like perceptual learning and plasticity (Klink et al. 2010, Lunghi and Sale 2015), inhibition (Lunghi et al. 2015, Ip et al. 2021), and stereopsis (Wolfe 1986, Wilson 2017). Especially the duration of transitions has been repeatedly viewed as an indicator of the excitation-inhibition disbalance in the visual system, with the amount of GABAergic inhibition being negatively associated with transition durations (Cao et al. 2016, Robertson et al. 2016, Mentch et al. 2019). This excitation-inhibition disbalance is thought to be altered in neurodevelopmental disorders and manifests in binocular rivalry as longer transition times (Casanova et al. 2013, Robertson et al. 2013, Said et al. 2013, Jusyte et al. 2018, Dunn and Jones 2020). Our demonstration that transitions in binocular rivalry appear in different forms raises the question of whether all transition types are equally affected in neurodevelopmental disorders, or just one. Also, it remains unclear whether just the duration of transitions is affected, or also their appearance. A more differentiated quantification of transitions should help to better pinpoint the exact differences between normal and altered rivalry parameters and potentially generate more precise biomarkers of these disorders.

Last but not least, our findings have implications for designing control condition for binocular rivalry experiments. The imitation of rivalry alternations (the so-called “replay”) is often used as a control condition to exclude observers that do not reliably report perception, to rule out any unspecific effects of experimental manipulation on rivalry reporting or to distill the unique neural signatures of rivalry in neuroimaging studies. Creating a realistic replay condition in binocular rivalry studies is always a challenging task. Since imitated transitions in replay are clearer and better defined in time, observers may report their replay experience more accurately than rivalry, which makes both conditions less comparable. Our results showing the variety of transitions during binocular rivalry can help to counteract this issue. The design of a replay condition could consider and try to mimic different transition types, making it more similar to the actual perception of the observers.

### Implications for neuroimaging studies

Although criticized for its artificial nature and reliance on behavioral report (Blake et al. 2014), binocular rivalry remains a key experimental paradigm for unravelling the neural mechanisms behind internally induced changes in perception (example recent studies include e.g. de Jong et al. 2020, Hesse and Tsao 2020, Weilnhammer et al. 2021, Kapoor et al. 2022, Lyu et al. 2022, Schauer et al. 2024). There is no doubt that binocular rivalry involves a substantial amount of competition at the early stages of visual processing (Leopold and Logothetis 1996, Polonsky et al. 2000, Tong and Engel 2001, Haynes et al. 2005). However, specifically the periods of subjective change in perception during binocular rivalry have been repeatedly linked to activity in frontal and parietal areas both in animals and humans with various neuroimaging methods (Lumer et al. 1998, Carmel et al. 2010, Zaretskaya et al. 2010, Britz et al. 2011, Knapen et al. 2011, Gelbard-Sagiv et al. 2018, Weilnhammer et al. 2021, Drew et al. 2022, Kapoor et al. 2022). Here we show that transitions may vary in appearance depending on the exact stimulus content used to induce binocular rivalry, and even from one transition to the other for the same stimulus content. Different transition appearance may lead to different neural activity patterns that are associated with it. A percept of randomly moving dots, for instance, will cause a different pattern of brain activity than a half-face half-house percept. A half-face half-house percept, in turn, will cause a different pattern of activity across the ocular dominance columns in V1 compared to a superimposed percept. Consequently, the observed brain activity may reflect the way transitions are perceived by the subjects rather than the fact of perceptual change. This, in turn, can lead to discrepancies in activation patterns reported by different studies and render them incomparable. To isolate the neural mechanisms underlying binocular rivalry transitions, it may be critical to create a control condition that is matched not only in transition duration but also in its appearance.

### Mechanisms underlying different transition types

Our findings suggest that different transition types may be mediated by different underlying mechanisms. In our study, we observed a dependency between the exact image content that induced binocular rivalry and the frequency of different transition types. While two of the most frequent transition types, piecemeal and superimposed transitions, appeared equally often for all stimuli, immediate transitions (i.e. instantaneous switches between exclusive percepts) were observed most frequently for moving dots. This result confirms previous reports that binocular rivalry between two stimuli that share the same resources (in this case the dorsal stream hierarchy) is stronger, leading to fewer intermediate percepts (Alais and Parker 2006). On the other hand, we did not observe the same effect for stimuli that should engage in the competition along the ventral stream. On the contrary, face-house stimuli showed the smallest proportion of immediate transitions in our study, which is not fully in line with the stream competition hypothesis. One reason behind the particularly strong rivalry between moving dots with many immediate percepts may be the unique property of moving stimuli to perceptually cancel each other, as observed in motion-induced blindness (New and Scholl 2018). Interocular competition in this case may be enhanced by the cancellation strength of the dots moving in opposite directions at the binocular level. In any case, our results suggest that if a study aims at minimizing intermediate percepts, moving stimuli may be the best approach.

Interestingly, a combination of moving dots and a static face induced transition types related to center-surround organization of the visual field (transition types: center/surround, circular wave, dispersing/gathering, superimposed dispersing). This could reflect differences in the extent of foveal representation between the face-selective and motion-processing areas: receptive fields of face-selective areas are typically foveal or parafoveal (Hasson et al. 2002), whereas motion-selective areas typically have an extended representation of the peripheral visual field (Stephen et al. 2002). This could explain why transitions were described by our subjects as “Face was always associated with dots around the face” (S002, session 1, DI, center/surround), “Face would appear in the middle and spread outwards” (S007, session2, DI, dispersing/gathering), and “Face in the middle and sparkling dots around” (S033, session 1, DI, center/surround). The fact that such center-surround transitions were not observed for rivalry between face and grating stimuli confirms that they may be specifically related to the competition between the content of the visual field at the center and at the surround.

### A multidimensional space of perceptual transitions

**Figure 9:**
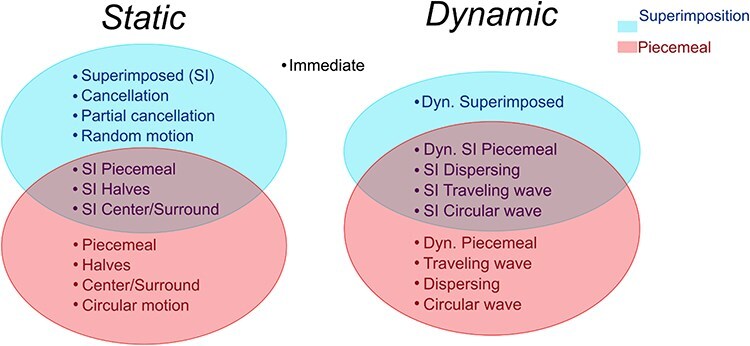
Multidimensional space of transitions in binocular rivalry. Transition categories were placed in a Euler diagram as categorized by piecemeal, superimposition, and dynamism.

Our categorization procedure thus revealed 20 different transition types. However, some of these types appear to represent composite types containing two elemental categories (e.g. “superimposed piecemeal”, consisting of “superimposed” and “piecemeal”). Furthermore, some transition types have a static and a dynamic version (e.g. superimposed vs. dynamic superimposed). We intentionally segregated such intermediate transitions into separate categories, mainly because participants themselves indicated such transitions as a separate category in the questionnaires. However, the existence of composite transitions suggests that the processes underlying certain transition types may act in parallel and that there may be some potential for reducing the number of categories, uniting them into category clusters. For example, halves and center/surround may be special cases of piecemeal transitions. In turn, “traveling wave” and “dispersing/gathering” can be dynamic versions of “halves” and “center/surround”. Moreover, “circular wave” may be a special case of “dynamic piecemeal”. Finally, one might argue that circular and random motion of dots is a subtype of superimposed or piecemeal transition. Cancellation and partial cancellation might also be a subtype of superimposed transition. Uniting the categories into larger clusters under consideration of the dynamic aspect yields a 3D feature space capable of describing the majority of transitions. This feature space is illustrated as a diagram in Fig. 9. Since the observer does not perceive anything during “immediate” transitions, they may be placed outside of the diagram. Crucially, even if reducing the number of categories to a few large category clusters, we still end up with a feature space with at least three dimensions with five or more clusters, underscoring the complexity of subjective visual experience during binocular rivalry.

### Limitations

Although our study demonstrated reliability and accuracy of the observer reports, there are several limitations of the methodology we used which future studies may consider.

First, in this study, the perceptual transitions were categorized based on the descriptions of individual untrained observers. Therefore, we should acknowledge that the variability between observers in their descriptions (be it verbal or a drawing) could have contributed to overestimating the final number of transition categories. Themes, which are created by raters during content analysis to “standardize” the descriptions across observers helped us to partially counteract this issue. Still, we cannot rule out that some of the inter-individual variability in descriptions remained unaccounted for. Future studies may try to approach this problem with few well-trained observers to see if it results in less categories. In fact, this has been an approach used to study the phenomenology of conscious experience during metacontrast masking (Koster et al. 2020). This approach, on the other hand, will be limited in assessing individual differences in perception. Overall, disentangling individual differences in subjective experience from the individual differences in the “description” of this experience remains an unresolved issue in consciousness research.

Another limitation of our method is the reliance on participants’ memory about multiple transitions over 4 min of binocular rivalry. In this period, the observers experience tens of transition episodes and are unlikely to be able to recall every one of them accurately, making their overall reports after each rivalry block less precise. Furthermore, these reports may be subject to recency effects and are therefore biased to the last few transitions. Further studies may be able to address this issue by using shorter rivalry trials with only a few transitions and collecting introspective reports after each of them.

Finally, the categories of perceptual transitions reported here might reflect the specific characteristics of our stimuli: gratings, images of house and face, and moving dots. Our stimulus choice was dictated by the common stimulus types used in binocular rivalry studies. It is very likely, however, that using other types of stimuli and their combinations would yield more or different transition categories. Furthermore, it is well known that the low-level features such as luminance, contrast and size of stimuli, spatial frequency of gratings, or speed of moving dots affect the duration of dominant percept, transition rate, and periods of exclusive perception, and, correspondingly, transition durations (Blake et al. 1992; Levelt 1965; Klink et al. 2010; Naber et al. 2020; Skerswetat et al. 2016). These parameters may have an influence on the appearance of perceptual transitions as well. Finding whether and how these stimulus parameters affect the appearance of transitions could be one of the future research directions.

## Conclusion

In conclusion, future studies on binocular rivalry may need to consider different transition appearances, which could yield new insights into the complexity of subjective visual experience and its neural mechanisms.

## Supplementary Material

niaf004_Supp

## Data Availability

Behavioral data and analysis code used in this experiment is publicly available on the Open Science Framework at doi: 10.17605/OSF.IO/N6DQF (Yilmaz and Zaretskaya 2024).
