# Supplementary material for: The complexity of human subjective experience during binocular rivalry
Source: Neurosci Conscious. 2025 Feb 28;2025(1):niaf004. doi: 10.1093/nc/niaf004 (PMC11879079; doi:10.1093/nc/niaf004)
Supplement: niaf004_Supp [file niaf004_supp.zip › suppl_data/Resubmission_SI_clean.docx]

# Supplementary Materials

## **Coding System for Categorization of Transition Descriptions (Smith, 2000)**

1. Research question and goal

Our research is categorization of transition appearance on binocular rivalry.

2. Methodology

We use content analysis to analyze the transition descriptions and the drawings of transition appearances, described and drawn by observers on a questionnaire (see Material).

3. Material

Our material for content analysis is the open-ended questionnaire including a question about the number of transition types the observer perceived during the corresponding block, the description(s) of the actual appearance(s) of transition(s), and a template(s) for drawing(s). The questionnaire solely relies on self-report.

4. How to select chosen material and its amount

We’ve chosen the questionnaire (see Material) for a data-driven search to define different transition types. According to the power analysis, we need data from at least 36 observers to investigate the transitions of 6 different stimulus pairs on health adults.

5. Which content analysis / coding system will be used

There is no ready-to-use coding system for our purpose. Therefore, we start with the transition types that were previously mentioned in the literature: halves, piecemeal, superimposed, and traveling wave. Moreover, immediate is an obvious category to us, considering the possibility of perceptual switch without any mixed percept. There are no further pre-defined categories.

6. Pilot with the method

We used the complete dataset of 5 observers as pilot data. We confirmed that the material is efficient for content analysis.

7. Train coders

Two experimenters worked together on the data of 30 observers during the construction of coding system. The data of first 5 observers is used as initial training dataset and then, the data of 30 observers is categorized to test the interrater reliability.

8. Real data recruitment

Data recruitment continue alongside of the analysis since our goal is to construct the current coding system.

9. Coding the data

The data were anonymized and the raters were agreed on the procedure.

10. Analysis

The final analysis was performed by the third experiment, and additional quantitative analysis will be performed to analyze the different transition types.

11. Interpretation

The interpretation of the results can be found in the manuscript.

Definition of units

• Text unit: questionnaire

• Coding unit: individual transition types

• Theme: keywords for each coding unit

• Context unit: every questionnaire of the same subject

### *Categories*

| Code | Subcode | Name | Description | Themes |
| --- | --- | --- | --- | --- |
| -1 | -1.0 | Remove data | When the participant described the dominant image as a transition category, we will exclude the data during quantitative analysis. |  |
| 0 | 0.0 | Immediate | There is no mixed percept between two dominant percepts. Subject’s percept changes immediately from one stimulus to another. The duration of transition is zero-second. | switch, immediate, sudden, abrupt, no mixed percept  Sketch: no drawing / drawing only one dominant image |
| 1 | 1.0 | Superimposed | Both stimuli are seen fully during the whole transition. The stimuli are superimposed with each other. There is no change in transparency. | overlapped, overlayed, both at the same time, blending (e.g., chess board for gratings)  Sketch: both stimuli are drawn as overlapped |
| 2 | 2.0 | Superimposed piecemeal | One stimulus is perceived either fully (or partially), the other stimulus is perceived partially. Only some parts are superimposed. | partially overlapped, both are visible at some parts  Sketch: some parts are drawn as overlapped |
|  | 2.1 | Superimposed halves | One stimulus is seen fully. Half of the other (top/bottom or left/right) is overlapped. | full stimulus plus one-half another stimulus, stimulus with top/bottom / left/right half the other stimulus  Sketch: one of stimuli is drawn as overlapped with the other only at one half |
| 3 | 3.0 | Piecemeal | Some random parts of the percept are from one stimulus, some parts are from the other stimulus but they don`t overlap. | fragment, partially seen, (partly one stimulus - partly other), (e.g., few random dots)  Sketch: some parts of both images are drawn |
|  | 3.1 | Halves | One half of the percept is one stimulus, the other half is the other stimulus. Mid-line can be either clear or blurry, stimuli may slightly overlap at mid-line. | half, [one] at bottom/top, [one] at right/left  Sketch: Half of the template is drawn by one stimulus, the other is by the other stimulus. |
|  | 3.2 | Center/Surround | One stimulus or part of stimulus in center, other stimulus is perceived around it/ at surrounds. Any parts don`t overlap. | [one] in center/ middle, [one] at surrounds/ around it/ at background  Sketch: one stimulus is drawn at the center and the other is drawn at surround |
| 4 | 4.0 | Dynamic superimposed | Both stimuli are seen fully during the whole transition. The transparencies of stimuli change during the transition. One gets more apparent and the other gets more transparent. | change in clarity, change in transparency, blending, “both visible, overlapping more and more, slow transition”  Sketch: both images are drawn as superimposed |
| 5 | 5.0 | Dynamic superimposed piecemeal | Similar to “superimposed piecemeal”. The subject perceives wider superimposed parts at random locations. Stimuli may be overlapped at the end. | partially overlapped, both are visible at some parts, different parts become visible |
|  | 5.1 | Superimposed traveling wave | One stimulus gets transparent starting from one edge or corner, like a curtain. Stimuli may be overlapped at the end. | invading from one side, overlapping from one side/ edge, laying on top of [one stimulus] from one side |
|  | 5.2 | Superimposed dispersing/gathering | One stimulus gets transparent and superimposed with the other starting from center outwards or from surrounds inwards. Stimuli may be overlapped at the end. | overlapping/ covering [one stimulus] starting from center/ surrounds, both visible, one stimulus on top of the other |
| 6 | 6.0 | Dynamic piecemeal | Similar to “Piecemeal”. The stimulus appears at more random locations or the apparent parts get wider. | changing parts, different parts become visible |
|  | 6.1 | Traveling wave | One stimulus takes over the other like a curtain, starting from one edge or corner. | invading from one side, curtain, “building” up, (“taking over” from edge) |
|  | 6.2 | Dispersing/Gathering | One stimulus is taking over the perception and replacing the dominant stimulus from the inside outwards or from the outside inwards. | appearing from center, invading from surround, (taking over/ from center/ surrounds) |
|  | 6.3 | Circular wave | One stimulus takes over the other one in a circular (clockwise or counter-clockwise) motion. | taking over in circular motion, circular curtain |
| 7 | 7.0 | Random motion of dots | *Only for Moving Dots.* Dots seem to move randomly. | chaos, random, fighting trembling |
| 8 | 8.0 | Cancellation | When two stimuli cancel each other. The participant does not see any stimuli but only the grey background or any motion but only static dots on the screen. | for dots: “pulsing” for gratings: “only gray background” |
|  | 8.1 | Partial cancellation | When two stimuli cancel each other partially. | for dots: “some dots don’t move”  for gratings: e.g., “X-shaped lines” |
| -2 |  | Undefined themes | When the raters decide to add a new category, they must write a clear definition and assign a few themes to the corresponding category. Exact naming and details for its definition will be decided with the other raters. |  |

### *Rules*

1. If the observer cannot define any mixed percept, the category is directly “immediate”. Do not confuse it with the dominant percept when the participant describes them separately on the questionnaire.

2. When it is superimposed (as illustrated in Figure 1a) and transparency is changing, the transition is dynamic. Otherwise, it is static as if transition is a third image in rivalry.

3. For moving dots, both motions can be visible in superimposed transition. The observer may interpret this percept as a combined motion as represented in the Figure 1b.

4. Sometimes the participant might describe the transition as “one stimulus being at background”. However, it can mean “at surround”. If it means “at surround”, it is center/surround. If it means “back of one stimulus”, it is superimposed. Do not confuse such cases. The drawing can help or better, the experimenter can ask details during the session and take notes.

5. Superimposed piecemeal is a mix type of superimposed and piecemeal categories. The superimposition occurs only at some parts of visual field.

6. Drawings for piecemeal, dynamic piecemeal, superimposed piecemeal, and dynamic superimposed piecemeal might be very similar. The description text helps for the actual categorization in such cases. The experimenter should be careful during the session (see Figure 2).

7. The halves can be interpreted as a new motion in Dots experiment as a linear motion (see Figure 3b). We still consider such cases as Halves.

8. Traveling wave does not have to start from an edge or a corner. The subject might define it by starting from a halves state. In such cases, it is considered still as traveling wave.

9. Similarly, dispersing/gathering might start from a state like center/surround. In such a ces, it is considered as dispersing/gathering.

10. If the dynamic change is defined different than how it is explained in Themes section, re-consider the category.

11. If the coding unit does not fit any of these categories, consider adding a new category, divide one into two categories or combine two of them.

12. When the description does not mention about the dynamism and explains the transition as a static image, choose the corresponding static category, e.g., static piecemeal, even if the participant selected the box “Dynamic”.

13. If the text unit is a questionnaire of replay session, avoid defining new categories and try to stick with superimposed if it is possible. However, do not bias!

Procedure

1. Start with the questionnaire. Go through each coding unit.

2. On the Excel sheet prepared for analysis, take Notes about every transition type. These are themes for our analysis.

- summarize the description

- use keywords to describe a transition type

Important: Take the Themes in Table as example. Using the same keywords and phrases from the Themes might be helpful for further categorization. However, limiting yourselves with those words might cause loss of some aspects. Thus, you can use different phrases if needed.

3. Take a look at every questionnaire of an observer. At the end of all three sessions, if there is a pattern which enhances the coding, use this information and modulate the Notes; e.g., the observer uses the word “sudden change” to define a brief transition whereas some use it for immediate. Then, change your Notes and make them “static transition” or something appropriate.

4. Categorize the transition appearance by comparing Themes in this guide and Notes that you.

Important: If you cannot find an appropriate category, either the summarizing was weak or it is a potentially new category. First, go back to the coding unit and try to improve your coding. If it does not solve the problem, take a note for undefined themes and try to suggest a new category.

## **Supplementary Table**

**Supplementary Table S1.** The parameters of the fit and the results of goodness-of-fit for the transition and dominance durations

| Stimulus pair | Dominance | BIC | Chi^2^ | df | p value |
| --- | --- | --- | --- | --- | --- |
| GG | Dominant | 3942 | 169 | 43 | < 0.001 |
| GG | Transition | 2947 | 119 | 42 | < 0.001 |
| II | Dominant | 2938 | 91 | 40 | < 0.001 |
| II | Transition | 3175 | 122 | 37 | < 0.001 |
| DD | Dominant | 3617 | 285 | 38 | < 0.001 |
| DD | Transition | 1570 | 218 | 37 | < 0.001 |
| GI | Dominant | 3190 | 67 | 43 | < 0.05 |
| GI | Transition | 3517 | 185 | 41 | < 0.001 |
| DI | Dominant | 3078 | 79 | 42 | < 0.001 |
| DI | Transition | 3129 | 154 | 39 | < 0.001 |
| DG | Dominant | 4305 | 108 | 48 | < 0.001 |
| DG | Transition | 2268 | 115 | 46 | < 0.001 |

## **
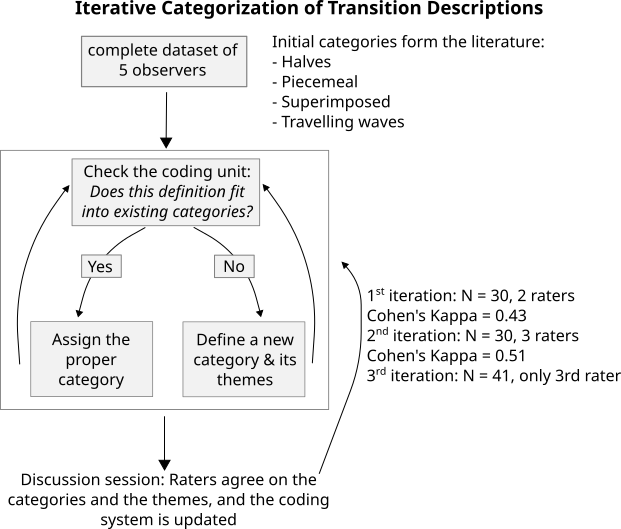
Supplementary Figures**

**Supplementary Figure S1.** Categorization workflow. The initial coding system was created based on the four transition types mentioned in the literature, namely halves, piecemeal, superimposed, and traveling wave. The data of the first 5 observers were used as a training dataset. Two raters defined themes and assigned categories for each coding unit, i.e., transition description. Then, the raters compared their evaluations in a discussion session and updated the coding system. During the first iteration, two raters completed the categorization of another 25 observers (N = 30 in total). They compared their categorization in the second discussion session and agreed on the final categorization. During the second iteration, a third rater followed the same procedure for the same 30 observers. Since the coding system was evaluated as moderately reliable, the third rater completed the categorization of the remaining data.

**Alt text:** A diagram showing the steps of categorization process. It includes 3 iterations to create a coding system.


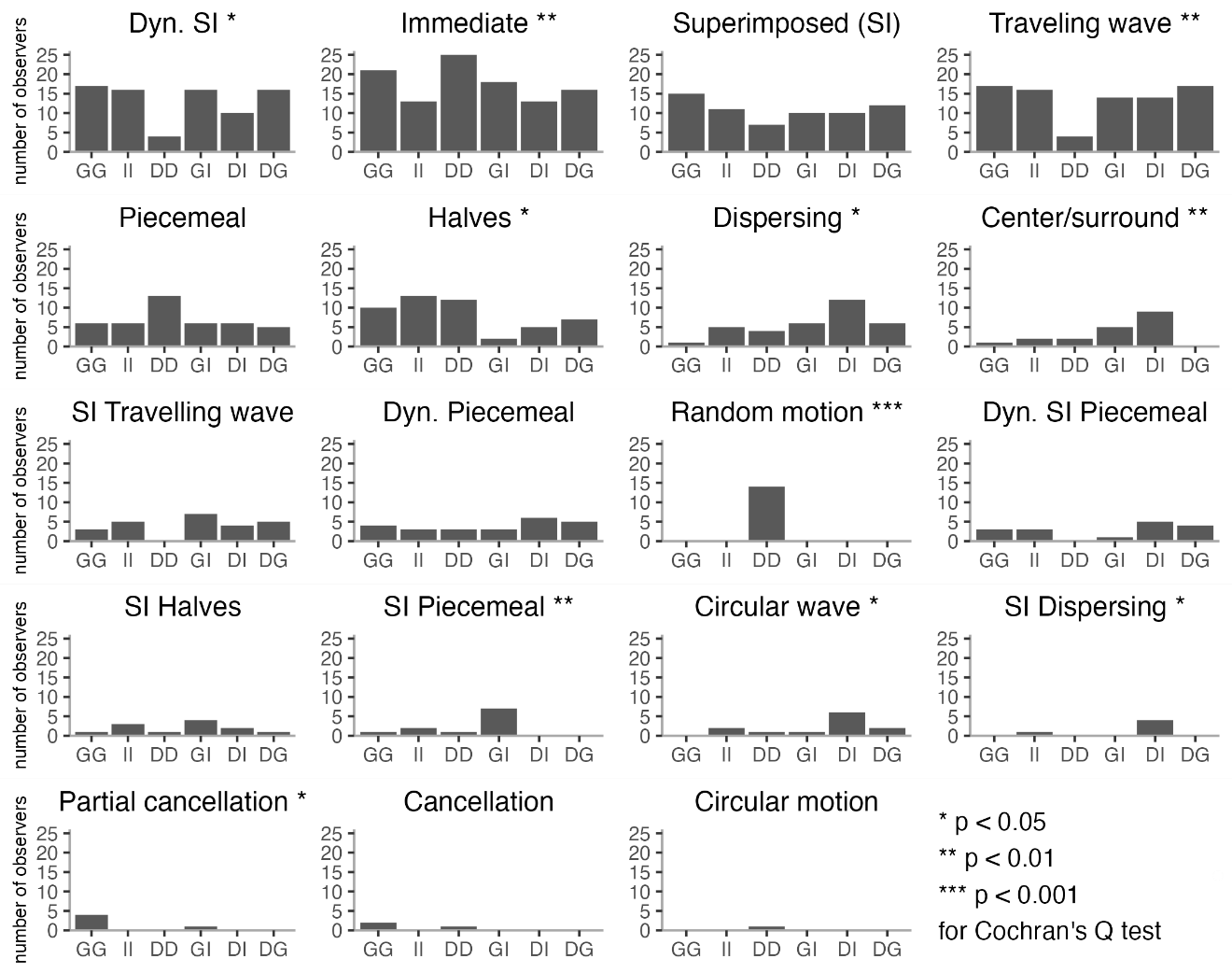


**Supplementary Figure S2.** Frequency of each transition category for each stimulus pair in session 1. The graphs are ordered according to the overall frequency of categories as shown in Figure 4. Asterisks indicate the significant effect of stimulus pair according to Cohran’s Q test. *: pfdr < 0.05, **: pfdr < 0.01, ***: pfdr < 0.001.

**Alt text:** Bar graphs showing the number of observers that described each transition type for each stimulus pair.


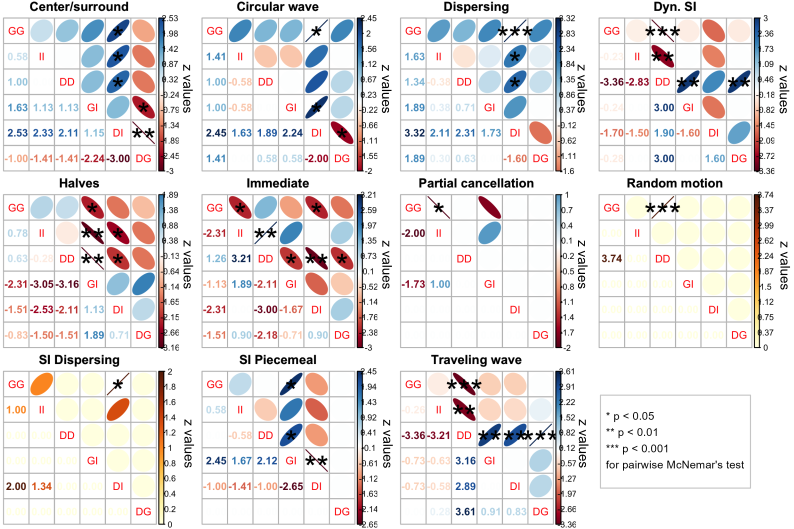


**Supplementary Figure S3.** Pairwise comparisons of frequencies for transition categories across stimulus pairs. *: p < 0.05, **: p < 0.01, ***: p < 0.001 (uncorrected).

**Alt text:** Visualization of the statistical results for pairwise comparisons in a matrix form. At diagonal, the stimulus pair labels are written. Upper part of the diagonal presents oval shapes of which color indicates the z values and size indicates p values. At the lower part of the diagonal, the z values were written.


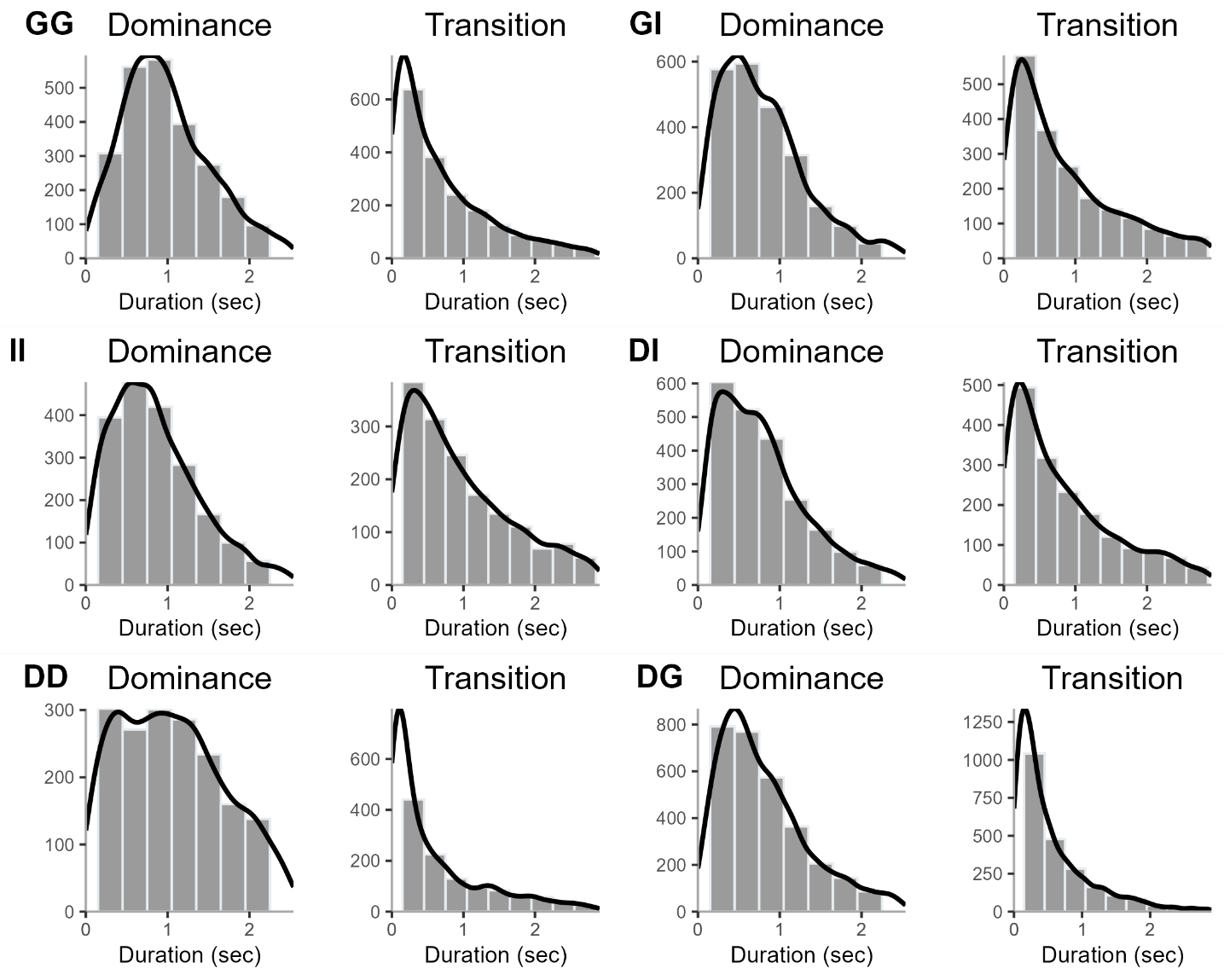


**Supplementary Figure S4.** Distribution of dominance and transition durations. We normalized the durations by the mean duration per observer to fit the data with a gamma distribution. Goodness-of-fit test shows that the duration of dominance and transition follows gamma distributions for all stimulus pairs (p < 0.05).

**Alt text:** Multiple graphs with histograms of dominance and transition durations. The data follow a skewed gamma-like distributions.


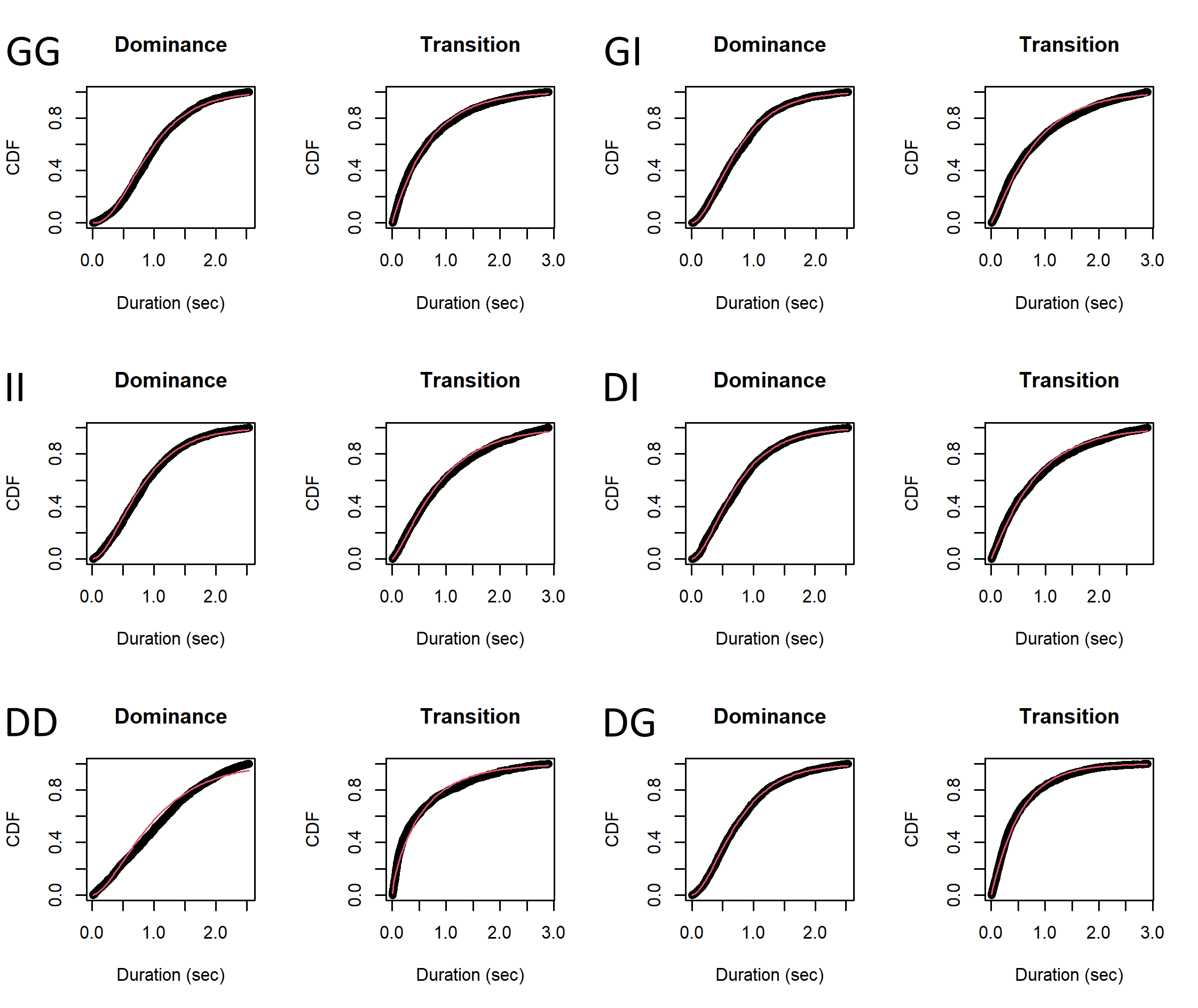


**Supplementary Figure S5.** Empirical (black) and theoretical (red) cumulative distribution functions (CDFs) of the fitted gamma distribution for dominance and transition durations.

**Alt text:** Graphs showing the gamma fit to the durations of dominant percept and transitions separately for each stimulus pair.

## **SI References**

Smith, C. P. (2000). Content Analysis and Narrative Analysis. In H. T. Reis & C. M. Judd (Eds.), Handbook of research methods in social and personality psychology (pp. 313–335). Cambrige University Press.
